# Effects of acute and chronic heat stress on the rumen microbiome in dairy goats

**DOI:** 10.5713/ab.24.0120

**Published:** 2024-06-26

**Authors:** Min Li, Lian-Bin Xu, Chen Zhang, Pei-Hua Zhang, Sha Tao, Hong-Yun Liu

**Affiliations:** 1 College of Animal Sciences, Zhejiang University, Hangzhou, 310058, China; 2 College of Animal Science and Technology, Hunan Agricultural University, Changsha, 410128, China; 3 Department of Animal and Dairy Science, University of Georgia, Athens, GA 30602, USA

**Keywords:** Acute, Chronic, Dairy Goats, Heat Stress, Microbiota, Metabolism

## Abstract

**Objective:**

The objective of this study was to reveal the influence of acute and chronic heat stress (HS) on the abundance and function of rumen microbiome and host metabolism.

**Methods:**

Forty mid-lactation goats were randomly divided into two artificial environments: control group and heat-stressed group. This study was recorded from two periods, 1 day and 28 days. The first day was defined as control 1 (CT1) and HS 1 (acute HS), and the last day was defined as CT28 and HS28 (chronic HS). On the first and last day, 6 dairy goats in each group were randomly selected to collect rumen liquid after the morning feeding through oral stomach tubes. The barn temperature and humidity were recorded every day.

**Results:**

Disruption of the rumen microbiome was observed under chronic HS, represented by an increase in the abundance of *Prevotella* and *Bacteroidales* (p<0.05), and upregulation of carbohydrate transport and metabolism functions (p<0.05). Additionally, the abundance of *Succinimonas* and *Ruminobacter* in chronic HS is lower than in acute HS (p<0.05), and the functions of intracellular trafficking, secretion and vesicular transport, and the cytoskeleton were downregulated (p<0.05).

**Conclusion:**

The HS affected the interaction between the microbiota and host, thereby regulated milk production in dairy goats. These findings increased understanding of the crosstalk between hosts and bacteria.

## INTRODUCTION

Heat stress (HS) is a negative condition characterized by a physiological strain in which the heat load surpasses the heat dissipation capacity of the body [1]. Dairy goats can adapt to climatic conditions, due to their large sweat glands and salivary glands [2]. Nevertheless, prolonged exposure to high temperatures decreases dry matter intake and milk yield [3], leads to animal discomfort and economic losses; and further causes imbalances in energy and nitrogen metabolism in dairy goats. A previous study revealed short-term HS diminished feed intake and milk production, long-term HS impaired rumen metabolism and mobilization of adipose tissue [4]. The temperature–humidity index (THI) is an indicator of heat stress for both humans and animals. Monitoring and controlling the THI can reduce the occurrence of heat stress and implement corresponding preventive measures [5].

The rumen has a high density of microorganisms including bacteria, archaea, protozoa, and fungi [6]. The microbial composition significantly changes under elevated temperature and humidity and is accompanied by decreased concentrations of short-chain fatty acids (SCFAs) [7]. Moreover, HS alters the population levels of specific bacterial groups in the rumen microbial community [8], and affects metabolic function in goats [9]. However, whether alterations in microbial structure and function under acute HS or chronic HS further affect milk performance is unclear.

Our previous study has indicated that chronic HS, com pared with acute HS, results in significantly lower milk yield, milk protein, and feed intake in dairy goats (unpublished data). The current study further compared the bacterial composition and functional enrichment terms in dairy goats exposed to acute and chronic HS and explored the interactions between the rumen bacteria and host. Our findings provided a new understanding of the interactions between the rumen bacteria and host and emphasized the importance of the co-development of the host and its microbiome.

## MATERIALS AND METHODS

### Animals and experimental design

The experiment was approved by the Animal Care Committee of Zhejiang University. The forty mid-lactation Saanen goats were randomly divided into two artificial environments: control group and heat-stressed group according to milk yield, body weight, and parity. The experiment consisted of a 1-week acclimatization period and a 4-week data-collection period. Data from two periods, after 1 day and 28 days of control and HS, were collected. The first day was denoted control (CT) 1 and HS1, and the last days were denoted CT28 and HS28. All animals had free access to drinking water and feed. These goats were provided with total mixed rations (TMRs) to ensure they have sufficient feed immediately after milking. The barn temperature and humidity were recorded at 8:00, 12:00, and 17:00 daily. The CT group was maintained at 18°C to 21°C using air conditions, and the HS group was subjected to temperatures of 37°C using a large electric fan heater during the day and 30°C at night. Humidity was maintained at 40% to 60% for groups. The barn average THI values were 66.69±0.61 (CT1 group), 81.56±2.70 (HS1 group), 66.31 ±0.37 (CT28 group), and 87.37±1.15 (HS28 group). THI was calculated by using temperature (T, °C) and humidity (RH, %) with the following model [10]:


THI=(1.8×T)-(0.055-0.005×RH)×(1.8×T-26)

### Detection of rumen fermentation parameters

On the first and last day, 6 dairy goats in each group were randomly selected to collect rumen liquid after the morning feeding through oral stomach tubes. All samples were stored at −80°C and used to detect ammonia nitrogen (NH_3_-N) and volatile fatty acids (VFAs). The ammonia nitrogen concentrations of the 24 samples were measured with phenol-sodium hypochlorite colorimetric analysis (Biochrom, Cambridge, UK) [11]. The proportion of VFAs was determined with gas chromatography (TRACE 1300; Thermo Scientific, Milan, Italy) [12].

### Rumen microbial DNA extraction and sequencing

The total DNA of the rumen liquid was extracted with a QIAamp DNA Stool Mini Kit (Qiagen, Redwood City, CA, USA). The quality and quantity of DNA samples were assessed with a NanoDrop instrument (Thermo, Beijing, China). DNA was amplified with primers 341F and 806R, targeting the V3 to V4 regions of the 16S rRNA sequence. The PCR products were purified with Agencourt AMPure XP and sequenced with the HiSeq platform.

### The 16S rRNA gene sequencing analysis

The raw data was filtered, Tags were connected with FLASH (v1.2.11) [13], and operational taxonomic units (OTUs) (>97% similarity) were clustered with USEARCH (v7.0.1090) [14]. The alpha diversity (ACE and Simpson) was analyzed with Mothur (v.1.31.2) [15]. Beta diversity analysis was assessed with QIIME (v.1.80) [16]. The distances among samples were analyzed with principal co-ordinates analysis (PcoA) and partial leastsquares discriminant analysis (PLS-DA). Linear discriminant analysis effect size (LEfSe: https://huttenhower.sph.harvard.edu/galaxy/) was used to identify bacteria with significant differential abundance among the four groups. Linear discriminant analysis (LDA) scores >2 and p<0.05 were considered to indicate differential abundance. In addition, metagenome function was predicted with PICRUSt2 (v2.2.0-b) and clusters of orthologous groups of proteins (COG) levels 1 and 2.

### Statistical analysis

Rumen NH_3_-N and VFAs were analyzed statistically through the MIXED procedure in SAS (Version 8.1; SAS Institute Inc., Cary, NC, USA). Results presented for type III tests of fixed effects and least squares mean. The statistical model included day, treatment, day×treatment, block, and covariate. Rumen NH_3_-N, pH, and VFAs of CT1, HS1, CT28, and HS28 goats were analyzed according to the following model:


$$\text{Y}=\mu +{\text{b}}_{\text{i}}+{\text{c}}_{\text{j}}+{\text{bc}}_{\text{ij}}+\beta {\text{X}}_{\text{ij}}+{\text{d}}_{\text{k}}+{ɛ}_{\text{ijk}}$$

where Y is the observed values; μ_i_ is the overall mean; b_i_ is the fixed effect of the treatment; I = 1, 2; c_j_ is the fixed effect of the treatment days; j = 1, 28; ɛ_ij_ is the interaction fixed-effect of ith treatment by the jth day; βX_ij_ is the pre-experimental values; d_k_ is the random effect with k_th_ sequence; k = 1, 2, 3, 4, 5, 6, 7, 8, 9; ɛ_ijk_ is the experimental error.

Correlation analysis was performed with Spearman’s cor relation, and Cytoscape_v 3.9.1 was used to visualize the gene-bacteria networks and gene-microbial metabolite networks [17]. The value of p<0.05 was considered to indicate statistical significance.

## RESULTS

### Rumen fermentation parameters

Twelve mid-lactation dairy goats were selected. Compared with the CT1 and CT28 groups respectively, the concentrations of acetate and propionate were significantly lower in the HS1 group and HS28 group (p<0.05; Table 1). The HS1 group did not show significant differences in butyrate, isobutyrate, valerate, isovalerate, and NH_3_-N concerning the CT1 group (p>0.05). However, valerate and isovalerate concentrations were significantly lower in the HS28 group than in the CT28 group (p<0.05).

### Rumen bacterial compositions

**Figure 1:**
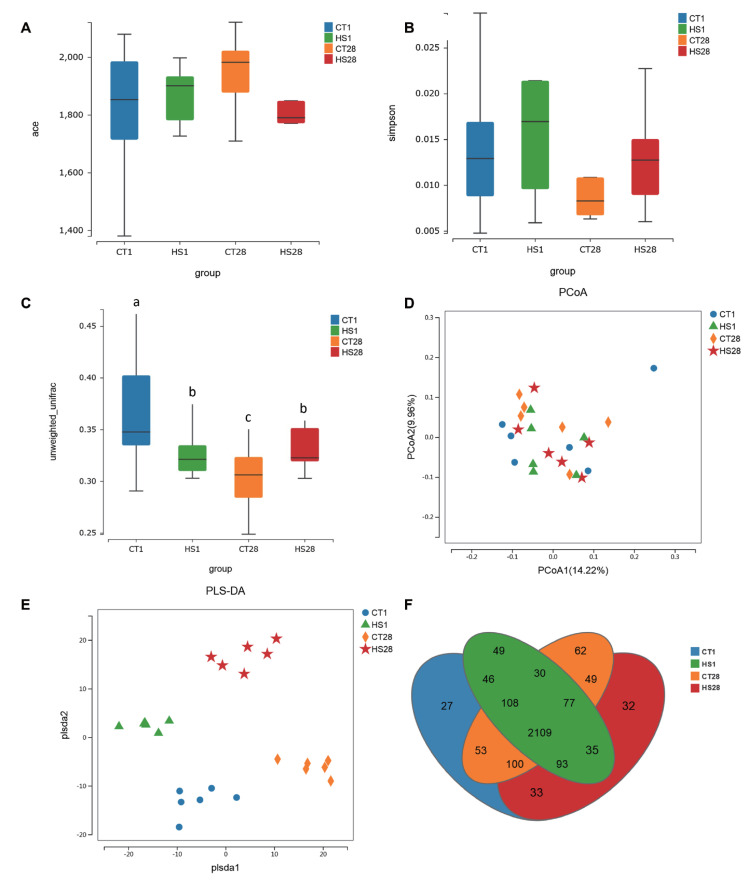
Acute and chronic HS altered the diversity of rumen microbiota in dairy goats. (A, B) The alpha diversity was estimated by using ace and simpson indices. (C)The Beta diversity of rumen microbiota. (D, E) PcoA and PLS-DA analysis of rumen microbiota. (F) Venn graph for OTUs among CT1 (blue), HS1 (green), CT28 (yellow), and HS28 (red) groups. PcoA, principal co-ordinates analysis; PLS-DA, partial leastsquares discriminant analysis; OTUs, operational taxonomic units; CT, control; HS, heat stress. ^a–c^ Means having different letters differ p<0.05.

A total of 3,684,647 raw reads and 3,653,368 clean reads were obtained in all samples. The ACE and Simpson indices did not significantly differ among the four groups (p>0.05; Figure 1A and 1B). Beta diversity illustrated the bacterial composition among the four groups (p<0.05; Figure 1C). In addition, the rumen microbiota was not separated according to HS1 treatment and HS28 treatment in PCoA (Figure 1D). Compared with CT treatment (CT1 and CT28), the rumen microbiota was separated by HS treatment (HS1 and HS28) in PLS-DA (Figure 1E). Moreover, the Venn diagram showed that 2,109 universal OTUs were detected among 2,903 total OTUs in all samples (Figure 1F). The *Bacteroidetes* (50.63% ±3.26%) and *Firmicutes* (39.20%±3.07%) were the predominant bacteria at the phylum level; *Bacteroidia* (42.23%± 2.88%) and *Clostridia* (31.03%±2.68%) were the predominant bacterial classes; *Prevotellaceae* (35.17%±2.71%) and *Ruminococcaceae* (13.11%±1.21%) were the predominant bacterial families; *Prevotella* (34.41%±2.68%) and *Ruminococcus* (33.63%±0.43%) were the predominant bacterial genera (Supplementary Figure S1A–S1F).

### Comparison of rumen bacteria by linear discriminant analysis effect size

**Figure 2:**
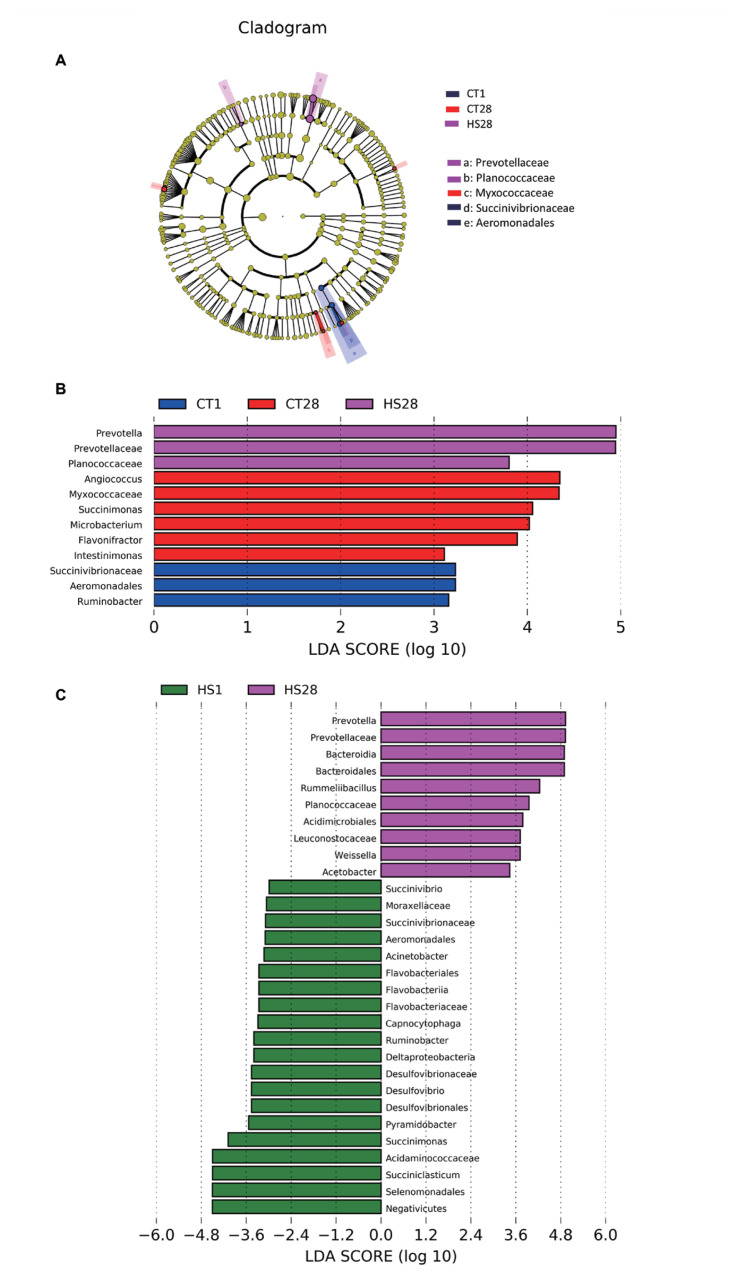
Comparison of the relative abundance of rumen bacteria among CT1 group, HS1 group, CT28 group, and HS28 group. (A) LEfSe taxonomic cladogram. Different colors represent biomarker taxon enrichment. (B) LDA score (log 10) of significantly different bacterial abundance. (C) LDA score (log 10) of the significantly different bacterial abundance of HS1 group and HS28 group. CT, control; HS, heat stress; LEfSe, linear discriminant analysis effect size; LDA, linear discriminant analysis.

The five clades as biomarkers were identified (Figure 2A). The LDA score indicated that HS28 treatment significantly increased the abundance of *Prevotella*, *Prevotellaceae*, and *Planococcaceae* (p<0.05), and CT28 treatment significantly increased the abundance of *Angiococcus*, *Myxococcaceae*, *Succinimonas*, and *Microbacterium* (p<0.05); HS significantly decreased the relative abundance of *Succinivibrionaceae*, *Aeromonadales*, *Succinimonas*, and *Ruminobacter* (p<0.05); and HS1 treatment did not significantly alter the relative abundance of rumen bacteria (Figure 2B). In addition, ten rumen bacterial species were significantly more abundant in the HS28 group than the HS1 group, including *Bacteroidia*, *Bacteroidales*, *Leuconostocaceae*, *Weissella*, and *Acetobacter* (p<0.05; Figure 2C).

### Effects of heat stress on metabolic pathways of the rumen microbiome

**Figure 3:**
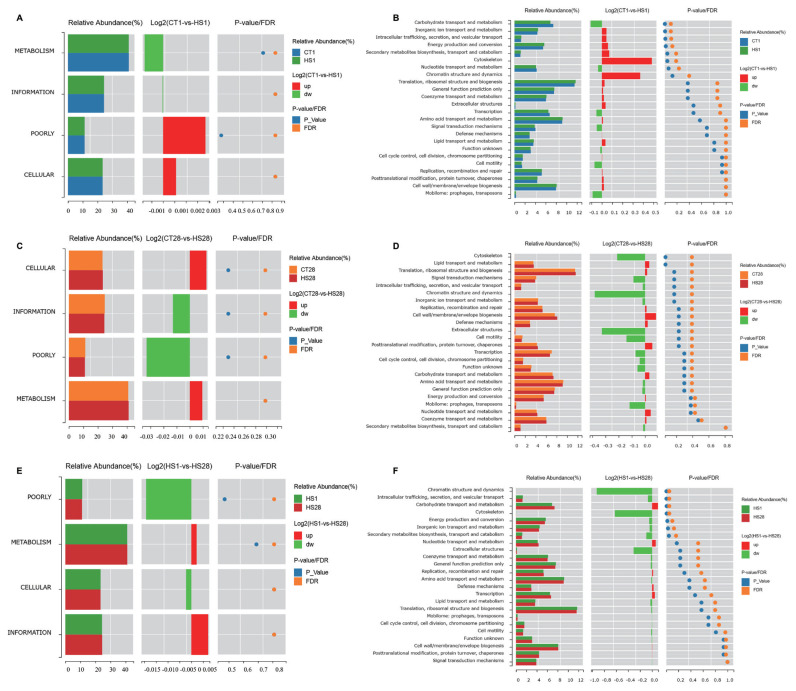
The metabolized pathways of different bacterial abundance after HS1 treatment and HS28 treatment in dairy goats at COG level 1 and level 2. HS, heat stress; COG, clusters of orthologous groups.

At clusters of orthologous groups (COG) level 1, the functions of poorly and cellular processing had an ascending tendency, but the metabolism and information showed a decreasing trend, in the HS1 group compared with the CT1 group (p> 0.05; Figure 3A). The cellular and metabolic functions showed an increasing trend after HS28 treatment (p>0.05; Figure 3C). Compared with those in the HS1 group, the metabolism and information showed greater enrichment in the HS28 group (p>0.05; Figure 3E). At COG level 2, carbohydrate transport and metabolism significantly were lower in the HS1 group compared with the CT1 group (p<0.05; Figure 3B). The lipid transport and metabolism were significantly enhanced, but the cytoskeleton was significantly diminished, by HS28 treatment compared with CT28 treatment (p<0.05; Figure 3D). In contrast to those in the HS1 group, the intracellular trafficking; secretion and vesicular transport; and the cytoskeleton were lower (p<0.05), but carbohydrate transport and metabolism were higher in the HS28 group (p<0.05; Figure 3F).

### Relationships of host genes with rumen bacteria and microbial metabolites

**Figure 4:**
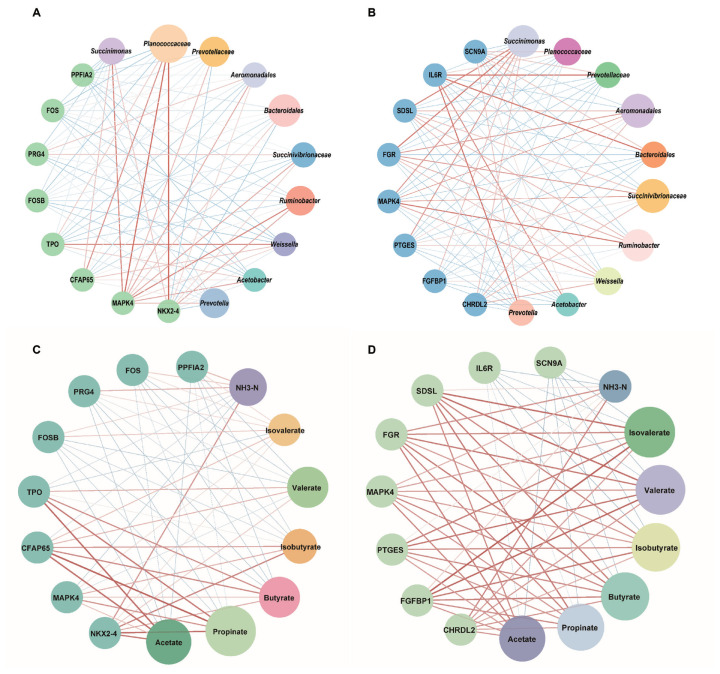
Co-occurrence between the ten different bacteria (acute HS(A); chronic HS(B)), microbial metabolites (acute HS(C); chronic HS(D)) and the eight differentially expressed genes (DEGs) under HS in dairy goats. Red edges respresent positive correlation, blue edges respresent negative correlation. Circles represent bacteria, metabolites and DEGs, and circles larger for bacteria or metabolites indicate stronger correlation. HS, heat stress.

A total of 48 up-regulated and 167 down-regulated differentially expressed genes (DEGs) were identified in acute HS, whereas 230 DEGs (80 up-regulated and 150 down-regulated) were observed in chronic HS (unpublished data). The 16 genes were selected from transcripts in dairy goats subjected to acute and chronic HS (Supplementary Table S1). The genes *NKX2–4*, *MAPK4*, *CFAP65*, and *TPO* were significantly downregulated in mammary gland (MG) tissues exposed to acute HS. *MAPK4* was positively correlated with *Succinimonas* and *Ruminobacter* (Pearson’s |*r*|>0.5; Figure 4A). Acetate and propionate were positively correlated with *NKX2–4*, *CFAP65*, and *TPO* (Pearson’s |*r*| >0.5; Figure 4C). However, *FOSB*, *PRG4*, *FOS*, and *PPFIA2* were upregulated and showed a weak relationship with bacteria.

Furthermore, six downregulated genes ( *CHRDL2*, *FGFBP1*, *PTGES*, *MAPK4*, *FGR*, and *SDSL*) and two upregulated genes (*IL6R* and *SCN9A*) were identified in MG tissues exposed to chronic HS. Meanwhile, *Prevotellaceae*, *Prevotella*, and *Bacteroidales* had a negative relationship with *CHRDL2* and *FGR*, but a positive relationship with *IL6R* (Pearson’s |*r*| >0.5; Figure 4B). Acetate, propionate, and butyrate had a positive relationship with *FGR* and *SDSL*, and a negative relationship with *IL6R* and *SCN9A* (Pearson’s |*r*| >0.5; Figure 4D).

## DISCUSSION

To date, studies have shown that HS had negative effects on feed intake and milk performance. In our study, rumen microbial composition and functional terms were compared, and the relationships of rumen bacteria with fermentation parameters and DEGs in dairy goats subjected to CT1, HS1, CT28, or HS28 treatments were analyzed. Chronic HS had more pronounced effects on ruminal composition and function than acute HS. Furthermore, different microbiota regulated body metabolism via co-development of microorganism-host system [18]. These findings might potentially guide the management of dairy goats to decrease the negative effects of high temperatures and improve overall health and productivity by targeting rumen microorganisms.

The results showed that the concentrations of acetate and propionate were higher in CT1 than in HS1 dairy goats, as well as higher in CT28 than in HS28 dairy goats, which indicated that HS1 and HS28 dairy goats may have less energy for lactation. However, there were no differences between HS1 and HS28 dairy goats, suggesting that the physiological metabolism might not significantly change once the temperature reached the threshold. The concentrations of acetate and propionate were higher in CT1 than in HS1 dairy goats, and in CT28 than HS28 dairy goats, thus indicating that HS1 and HS28 dairy goats may have relatively less energy for lactation [19]. The content of VFAs and NH_3_-N was affected primarily by the rumen microbiota. No observable differences in the value of Ace and Simpson indexes, as well as the total number of OTUs, as previously described 23 to 25. The beta diversity showed a lower bacterial diversity in HS1, CT28, and HS28 groups, indicating that the ruminal bacterial composition was changed and the diversity was reduced under high-temperature conditions. Chronic HS led to a significantly greater abundance of *Prevotella*, *Prevotellaceae*, and *Planococcaceae* than observed in the other groups. *Prevotella* is central to carbohydrate and hydrogen metabolism [20]. However, the presence of *Prevotella* has been associated with the development of inflammatory autoimmune diseases and opportunistic infections [21]. In contrast, *FGR* and *IL6* were highly associated with *Prevotella*, as well as acetate, propionate, and butyrate [22]. The strong relationship between the expression of genes and SCFAs indicated that the microbiota was likely to be associated with the transport and absorption of amino acids [23]. Chronic HS might lead to changes in gene expression in MG tissue, heighten the body’s inflammatory response, and ultimately diminish lactation performance in dairy goats [24]. Moreover, chronic HS led to a significant increase in the abundance of *Bacteroidales*, *Weissella*, and *Acetobacter*, all of which used carbohydrates or starch as an energy source. *Bacteroidales* were associated with biohydrogenation of fatty acids and fiber digestion [25]. Herein, *Bacteroidales* were negatively correlated with *CHRDL2* and *FGR*, and positively correlated with *IL6*, it suggested that rumen bacteria were involved in intrucate ecological and host interactions, and may potentially exert substantial indirect effects on milk production. Genes like *FGFBP1* and *PTGE* which related to cell proliferation, apoptosis, and immunity were necessary for sustained recovery capacity. Additionally, microbial correlations were observed, suggesting potential metabolic and immune responses to heat stress in dairy goats.

Chronic HS resulted in a significantly lower abundance of *Succinivibrionaceae*, *Succinimonas*, and *Ruminobacter* than acute HS. The microorganisms of *Succinimonas* and *Ruminobacter* are associated with energy metabolism and methane emission and are used by other microorganisms to synthesize propionate and valerate [26,27]. The diminished abundance of *Succinimonas* and *Ruminobacter* decreased starch degradation and the bioproduction of VFA, thus further affecting the absorption of mammary epithelial cells. MAPK4 protein directly activates AKT, thereby promoting cell migration in triple-negative breast cancer [28]. Interestingly, we compared the differential expression of genes involved in chronic HS in MG tissues. Therefore, downregulated *MAPK4* might inhibit mammary cell proliferation and decrease the secretion of milk constituents in dairy goats during HS. Moreover, *Succinimonas* and *Ruminobacter* were positively correlated with *MAPK4* expression, thus suggesting that microorganisms might regulate the expression of cellular genes through interactions or microbial metabolites. Furthermore, *NKX2–4*, *CFAP65*, and *TPO* were positively correlated with acetate and propionate, thereby supporting the effects of rumen microbiota-derived SCFAs on MG metabolism [29]. The correlation between microbial communities and gene expression suggests complex interactions between metabolic and immune responses to heat stress in dairy goats.

The study revealed that the metabolic of the rumen mi crobiome were affected by acute and chronic HS, such as chromatin structure and dynamics, intracellular trafficking, secretion, and vesicular transport. The pathways of carbohydrate transport and metabolism were diminished in acute HS but enriched in chronic HS, thus indicating that dairy goats with prolonged HS might have alterations in the basic physiological mechanisms and accelerated carbohydrate metabolism to satisfy energy requirements for maintenance and lactation. Therefore, the rumen bacterial function mirrors the mechanism by influencing phenotypes. However, the abundance and function of the rumen microbiome caused by acute and chronic HS, were direct or indirect, require further investigation. In addition, archaea, protozoa, and anaerobic bacteria play essential roles in ruminal metabolism, but we focused on bacterial genera. The functions of archaea, protozoa, and anaerobic bacteria in HS should be examined in future studies. Acute and chronic HS shifted rumen bacteria toward those associated with rumen fermentation and host metabolism. Chronic HS increased the abundance of *Prevotella* and *Bacteroidales* and decreased the abundance of *Succinimonas* and *Ruminobacter*. These findings revealed an important contribution of the rumen bacteria to host metabolism and suggested promising directions in manipulating the rumen bacteria to prevent energy supply deficiency. Moreover, HS-resistant bacteria might potentially serve as probiotics to mitigate the negative effects of HS to improve the welfare and productivity of dairy goats under challenging environmental conditions.

## Figures and Tables

**Table 1:** Effect of acute and chronic heat stress on rumen fermentation parameter

Items	Treatment				SEM	p-value		
	
	1 d		28 d			Treat	Day	Treat×day
	
	CT	HS	CT	HS				
Acetate (mmol/L)	33.29^a^	22.91^b^	39.09^a^	22.85^b^	2.12	<0.01	0.30	0.28
Propionate (mmol/L)	8.81^a^	5.85^b^	10.83^a^	5.73^b^	0.56	<0.01	0.23	0.18
Butyrate (mmol/L)	5.53^b^	4.75^b^	8.03^a^	3.74^b^	0.43	<0.01	0.10	<0.01
Isobutyrate (mmol/L)	1.23^a^	1.03^a^	0.57^b^	0.40^b^	0.09	0.08	<0.01	0.90
Valerate (mmol/L)	0.70^a^	0.67^a^	0.68^a^	0.38^b^	0.04	0.01	0.02	0.04
Isovalerate (mmol/L)	1.04^a^	1.01^a^	0.77^b^	0.46^c^	0.06	0.08	<0.01	0.13
NH_3_-N (mg/dL)	1.38^ab^	1.58^a^	1.33^ab^	1.31^b^	0.07	0.61	0.01	0.04
pH	6.50	6.54	6.28	6.33	0.05	0.71	0.05	0.94

CT, control; HS, heat stress; SEM, standard error of the mean.

a–c Value within the row of each variable is significantly different (p<0.05).

## SUPPLEMENTARY MATERIAL

**Supplementary Table S1**. Differentially expressed genes (p<0.01, |Log2FC| >1.2) in mammary gland tissue of dairy goats with acute HS and chronic HS

**Supplementary Figure S1**. The relative abundance of predominant bacteria after acute and chronic HS in CT1 group, HS1 group, CT28 group, and HS28 group were shown at phylum (A) class (B), order (C), family (D), genus (E), and species (F).
