# Supplementary material for: Effects of acute and chronic heat stress on the rumen microbiome in dairy goats
Source: Anim Biosci. 2024 Jun 26;37(12):2081–90. doi: 10.5713/ab.24.0120 (PMC11541016; doi:10.5713/ab.24.0120)
Supplement: Supplementary file 1 [file ab-24-0120-Supplementary-Table-S1.pdf]

Table S1. Differentially expressed genes (P<0.01, |Log2FC| >1.2) in mammary gland tissue of dairy goats with acute HS and chronic HS.

Gene	Description	Log2FC	*P* value
**HS1 VS CT1**			
NKX2-4	NK2 homeobox 4	-4.94	3.51E-05
MAPK4	Mitogen-activated protein kinase 4	-2.54	5.37E-08
CFAP65	Cilia and flagella associated protein 65	-2.39	1.80E-04
TPO	Thyroid peroxidase	-2.16	1.84E-05
FOSB	FosB proto-oncogene, AP-1 transcription factor subunit	2.42	1.71E-05
PRG4	Proteoglycan 4	2.81	5.33E-04
FOS	Fos proto-oncogene, AP-1 transcription factor subunit	2.96	1.55E-06
PPFIA2	PTPRF interacting protein alpha 2	3.24	1.35E-04
**HS28 VS CT28**			
CHRDL2	Chordin like 2	-1.62	0.001
FGFBP1	Fibroblast growth factor binding protein 1	-1.62	1.45E-06
PTGES	Prostaglandin E synthase	-1.56	4.43E-05
MAPK4	Mitogen-activated protein kinase 4	-1.48	2.70E-06
FGR	FGR proto-oncogene, Src family tyrosine kinase	-1.46	2.44E-05
SDSL	Serine dehydratase like	-1.43	4.44E-06
IL6R	Interleukin 6 receptor	1.23	1.03E-06
SCN9A	Sodium voltage-gated channel alpha subunit 9	1.36	3.94E-09
