# Supplementary material for: Effects of acute and chronic heat stress on the rumen microbiome in dairy goats
Source: Anim Biosci. 2024 Jun 26;37(12):2081–90. doi: 10.5713/ab.24.0120 (PMC11541016; doi:10.5713/ab.24.0120)
Supplement: Supplementary file 2 [file ab-24-0120-Supplementary-Fig-S1.pdf]

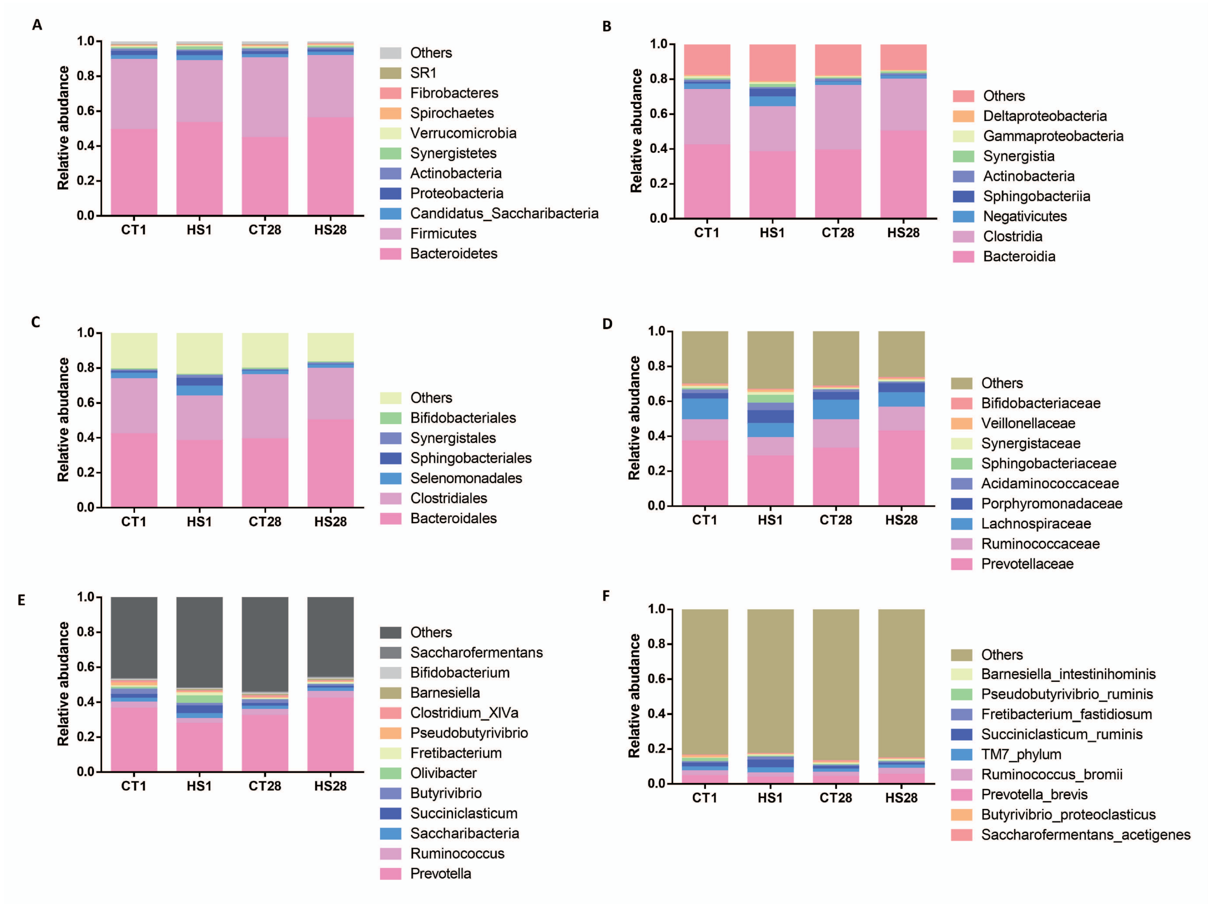


**Figure S1**. The relative abundance of predominant bacteria after acute and chronic HS in CT1 group, HS1 group, CT28 group, and HS28 group were shown at phylum (A), class (B), order (C), family (D), genus (E), and species (F).
